# Navigating the Cytokine Storm: A Comprehensive Review of Chemokines and Cytokines in Sepsis

**DOI:** 10.7759/cureus.54275

**Published:** 2024-02-15

**Authors:** Harshitha Reddy, Chaitanya Kumar Javvaji, Suprit Malali, Sunil Kumar, Sourya Acharya, Saket Toshniwal

**Affiliations:** 1 Internal Medicine, Jawaharlal Nehru Medical College, Datta Meghe Institute of Higher Education and Research, Wardha, IND; 2 Pediatrics, Jawaharlal Nehru Medical College, Datta Meghe Institute of Higher Education and Research, Wardha, IND

**Keywords:** biomarkers, therapeutic interventions, immune response, chemokines, cytokine storm, sepsis

## Abstract

This comprehensive review thoroughly explores the intricate relationship between chemokines, cytokines, and the cytokine storm in sepsis, offering a nuanced understanding of the molecular mechanisms underpinning this life-threatening syndrome. Beginning with examining sepsis stages and immune response dynamics, the review emphasizes the dysregulation leading to the cytokine storm, where pro- and anti-inflammatory cytokines disrupt the delicate immune equilibrium. Delving into chemokines, the discussion encompasses subfamilies, receptors, and functions, highlighting their critical roles in immune cell migration and activation during sepsis. The implications for clinical practice are substantial, suggesting avenues for targeted diagnostics and therapeutic interventions. The review identifies areas for future research, including the search for novel biomarkers, deeper insights into cytokine regulation, and the pursuit of personalized medicine approaches. This comprehensive exploration aims to guide clinicians, researchers, and policymakers in navigating the complexities of sepsis, fostering a foundation for transformative advancements in understanding and managing this formidable clinical challenge.

## Introduction and background

Sepsis, a life-threatening condition triggered by the body's extreme response to infection, remains a formidable challenge in modern medicine. The clinical manifestations of sepsis encompass a dysregulated host response to pathogens, leading to organ dysfunction and failure. Defined by the Sequential Organ Failure Assessment (SOFA) criteria, sepsis arises when the body's response to an infection injures its tissues and organs, culminating in a cascade of events that can prove fatal if not promptly addressed [1]. At the heart of sepsis pathology lies the intricate interplay of the immune system, where an exuberant release of cytokines, known as the "cytokine storm," plays a pivotal role [2]. This overwhelming immune response, initially intended to combat the invading pathogens, paradoxically contributes to widespread tissue damage and organ dysfunction. Understanding the nuanced involvement of cytokines in sepsis is imperative, as it not only shapes the disease's progression but also holds the key to developing targeted therapeutic interventions [3].

This comprehensive review aims to dissect the complexities surrounding the cytokine storm in sepsis, with a particular emphasis on the roles of chemokines and cytokines. By delving into this phenomenon's molecular and cellular underpinnings, we aim to provide a holistic understanding of the immunological landscape during sepsis. Furthermore, this review seeks to bridge the gap between current knowledge and emerging research, shedding light on potential avenues for therapeutic advancements. As we navigate through the various facets of chemokines and cytokines in sepsis, our goal is to offer insights that deepen scientific comprehension and inspire innovative approaches for mitigating the impact of sepsis on global health.

## Review

Overview of sepsis and cytokine storm

Sepsis, a multifaceted syndrome, unfolds in distinct stages that reflect the severity of the body's response to infection. The journey begins with infection, where the immune system recognizes and attempts to eradicate invading pathogens. However, when the immune response becomes dysregulated, sepsis ensues. The progression of sepsis is commonly categorized into stages, ranging from sepsis to severe sepsis and, ultimately, septic shock. These stages, often defined by clinical and physiological parameters, serve as crucial benchmarks for understanding the evolving landscape of the condition [4]. The immune response to infection is a finely orchestrated involving various cellular and molecular players. This can become discordant in sepsis, leading to a dysregulated immune response. Immune cells, including macrophages and neutrophils, play pivotal roles in recognizing and responding to pathogens. However, in sepsis, an imbalance in the activation and regulation of these cells can result in tissue damage, contributing to the overall pathogenesis of the condition [5]. At the core of orchestrating the immune response are cytokines, which are small proteins acting as messengers between immune cells. In the context of sepsis, the immune system frequently initiates an exaggerated cytokine response, commonly referred to as the cytokine storm. This sequence involves the release of pro-inflammatory cytokines, including Interleukin-1 (IL-1) and Tumor Necrosis Factor-alpha (TNF-α), leading to hyperinflammation. Concurrently, anti-inflammatory cytokines such as Interleukin-10 (IL-10) and Transforming Growth Factor-beta (TGF-β) are upregulated to counterbalance the inflammatory environment. The intricate balance between pro- and anti-inflammatory signals becomes disrupted in sepsis, exacerbating the systemic complications observed in affected individuals [6].

Chemokines- the signaling molecules

Introduction to Chemokines

Chemokines, recognized as chemoattractant cytokines, constitute a family of small proteins pivotal in cell signaling and modulation of immune systems. Their primary renown lies in their capacity to induce cell migration, particularly that of white blood cells. The definition of chemokines is rooted in their primary amino acid sequence and the specific arrangement of cysteines, forming disulfide bonds crucial for maintaining the chemokine monomer's structural integrity. Chemokines are classified into subfamilies, CC, CXC, CX3C, and XC, distinguished by the distinct configuration of the two cysteines nearest the N terminus. These proteins transmit signals through cell surface G protein-coupled heptahelical chemokine receptors. Virtually all diseases exhibit some involvement of chemokines and their receptors, with drugs targeting these receptors having successfully transitioned to clinical applications [7]. The integral role of chemokines in the immune system and their active participation in various diseases has undergone thorough scrutiny. Their well-established function as chemoattractants for immune cells has made them a prominent focus in scientific literature. The profound significance of chemokines in processes such as inflammation, infection, and cancer positions them as compelling subjects for potential therapeutic interventions [8]. As essential signaling molecules, chemokines intricately regulate immune cells' migration and spatial arrangement within the body. Given their multifaceted functions and implications in diverse diseases, chemokines remain at the forefront of extensive research, holding promise as potential targets for developing innovative therapeutic strategies.

Types of Chemokines

Chemokines, a prominent subfamily of small cell signaling proteins, also recognized as chemoattractant cytokines, play a pivotal role in the immune system by initiating and directing the recruitment and migration of immune cells. This diverse family is categorized into four main subfamilies: CXC, CC, CX3C, and XC. The classification is based on the arrangement of specific cysteines, pivotal in forming disulfide bonds crucial for maintaining the structural integrity of the chemokine monomer. Operating through cell surface G protein-coupled heptahelical chemokine receptors, these proteins serve as essential mediators in a spectrum of physiological and pathological processes. Notably, their involvement spans areas such as inflammation, infection, and cancer, rendering them subjects of extensive research and potential targets for therapeutic interventions [7,9].

Chemokine Receptors

Chemokine receptors, a specific type of cytokine receptor located on the surface of certain cells, engage in interactions with chemokines, a class of small proteins that hold fundamental significance in cell signaling and immune system modulation. Classified as a subset of G protein-coupled receptors (GPCRs), chemokine receptors exhibit a distinctive structure comprising seven transmembrane domains. This categorization places them within the extensive family of G protein-coupled receptors, emphasizing their role as integral signaling molecules. Presently, approximately 19 distinct chemokine receptors have been characterized. Despite sharing common structural features, such as a short and acidic N-terminal end, seven helical transmembrane domains, and an intracellular C-terminus containing serine and threonine residues serving as phosphorylation sites, these receptors exhibit selectivity in binding a limited number of ligands [10]. The engagement of chemokine receptors in the immune system is paramount, as they orchestrate the migration and positioning of immune cells in response to chemokine signals. This involvement extends across a spectrum of physiological and pathological processes, making chemokine receptors a focal point of extensive research and a potential target for therapeutic interventions [7].

Functions of Chemokines in Sepsis

Chemokines are crucial in sepsis, a life-threatening condition marked by an aberrant immune response to infection. In sepsis, chemokines contribute to the progression of organ damage and dysfunction. Notably, the chemokine interleukin-8 (IL-8) has shown elevated levels in patients experiencing septic shock, correlating with respiratory, renal, and hematological failure. Moreover, high circulating concentrations of chemokines can induce desensitization and suppress local inflammation. The involvement of chemokines in sepsis is an active area of research, with ongoing investigations into their potential as markers for assessing disease severity, organ dysfunction, and patient mortality [11]. In sepsis, the dysregulated immune responses, characterized by an elevation in cytokines and chemokines, contribute to the onset of a cytokine storm. This heightened immune response, often associated with the severity of the condition, further exacerbates organ damage and dysfunction in sepsis [12]. The dysregulation of the immune response, coupled with the cytokine/chemokine storm, holds significant implications for organ function and patient prognosis in sepsis [11,12].

Cytokines as key players in the cytokine storm

Overview of Cytokines

A cytokine storm represents a critical systemic inflammatory syndrome marked by heightened circulating cytokine levels and immune cell activation. Various factors can trigger this condition, including infections, cancers, autoimmune disorders, and genetic alterations. The dysregulated inflammatory response results in a substantial release of cytokines, leading to a self-sustained activation of immune cells and hyperinflammation, which can culminate in a life-threatening state. Infections of significant severity, such as sepsis, can escalate into an autoamplifying cytokine storm associated with adverse effects like organ damage and dysfunction. Acknowledging cytokine storm as a distinct entity is relatively recent, underscoring its importance for clinicians to identify due to its prognostic and therapeutic implications. While specific cytokines may aid in controlling infections, their excessive release can have deleterious effects. The pathophysiology, clinical consequences, therapeutic strategies, and prognosis of cytokine storms are actively researched areas. Innovative therapeutic approaches targeting the endothelium as both a source and target of cytokines are under exploration. Despite advancements in understanding the triggers of cytokine production, developing effective treatments targeting cytokines remains a formidable therapeutic challenge [13-15].

Pro-Inflammatory Cytokines

Pro-inflammatory cytokines, integral to the immune response and inflammation, are small secreted proteins generated in response to invading pathogens, stimulating, recruiting, and proliferating immune cells. Among the pivotal pro-inflammatory cytokines are Interleukin-1 (IL-1), IL-6, and Tumor Necrosis Factor-alpha (TNF-α) [16]. Interleukin-1 (IL-1): IL-1, a significant pro-inflammatory cytokine, plays a crucial role in immune response and inflammation, regulating various cellular processes, including cell growth, differentiation, and activation [16]. IL-1 further divides into IL-1α and IL-1β, each possessing distinct functions and regulatory mechanisms [17]. Tumor Necrosis Factor-alpha (TNF-α), another key pro-inflammatory cytokine, regulates immune responses, inflammation, and the development of various diseases. Produced by various immune cells such as macrophages, dendritic cells, and T cells, TNF-α plays a crucial role in coordinating cell-mediated immune responses [16]. Additionally, TNF-α can directly impact non-immune cells, including endothelial cells and fibroblasts, contributing to inflammation and tissue damage [17]. These pro-inflammatory cytokines transmit signals via type I cytokine receptors (CCR1), structurally divergent from each other [16]. The production and regulation of these cytokines are vital for the immune system's response to infections and other inflammatory stimuli. However, dysregulated production of these cytokines can lead to severe inflammation, contributing to various diseases, such as autoimmune disorders, allergic reactions, and sepsis [18]. Table 1 describes the pro-inflammatory cytokines as potential biomarkers in sepsis [16-18].

**Table 1:** Pro-inflammatory cytokines as potential biomarkers in sepsis

Biomarker	Role in Cytokine Storm	Diagnostic Significance	Clinical Challenges
IL-1β	Initiates and amplifies immune responses	Indicates sepsis severity	Translation to effective treatments faces challenges
CCL4	Chemotactic factor for immune cells	Biomarker for cytokine storms	Requires thorough validation for clinical use
CCL5	Regulates immune cell migration	Associated with sepsis severity	Bridging the gap between promising biomarkers and clinical efficacy
CXCL10	Induces chemotaxis in various immune cells	Reflects inflammatory milieu	Challenges in translating biomarkers to successful clinical treatments

Anti-Inflammatory Cytokines

Interleukin-10 (IL-10): Interleukin-10 (IL-10) is a cytokine renowned for its potent anti-inflammatory properties. Acting as a critical modulator of the immune response, IL-10 exerts its influence by inhibiting the production of pro-inflammatory cytokines, including but not limited to IL-1β, IL-6, and Tumor Necrosis Factor-alpha (TNF-α) [17]. In doing so, IL-10 helps temper the excessive activation of the immune system, preventing an overzealous inflammatory response. Furthermore, IL-10 plays a crucial role in fostering an anti-inflammatory environment by promoting the production of other anti-inflammatory cytokines, such as Interleukin-1 receptor antagonist (IL-1Ra) [17]. This dual action positions IL-10 as critical in maintaining immune homeostasis and preventing unwarranted inflammation.

Transforming growth factor-beta (TGF-β): Transforming Growth Factor-beta (TGF-β) stands out as a multifunctional cytokine with diverse effects, including anti-inflammatory, immunosuppressive, and profibrotic properties. In its anti-inflammatory role, TGF-β inhibits the production of pro-inflammatory cytokines, thereby contributing to the resolution of inflammation [17]. Additionally, TGF-β plays a pivotal role in tissue repair and regeneration, emphasizing its importance in orchestrating the healing process [17]. The intricate functions of TGF-β highlight its versatility in regulating immune responses and maintaining tissue homeostasis. However, the production and regulation of these anti-inflammatory cytokines, including IL-10 and TGF-β, can be intricate processes influenced by various factors. The interplay with pro-inflammatory cytokines and the specific context of the immune response add complexity to their regulatory mechanisms, underscoring the nuanced nature of immune modulation [17]. Despite their complexity, these anti-inflammatory cytokines remain essential components in the intricate balance required for the proper functioning of the immune system and the resolution of inflammation.

Dual-Function Cytokines

Interleukin-6 (IL-6): Interleukin-6 (IL-6), once perceived solely as a pro-inflammatory cytokine, has emerged as a multifaceted player in immune modulation. Its functions extend beyond inflammation, showcasing pro-inflammatory and anti-inflammatory properties contingent upon the specific context and receptors involved [19]. IL-6 exhibits pro-inflammatory activity when interacting with the classic IL-6 receptor (IL-6R), promoting inflammation as traditionally understood. Intriguingly, IL-6 can also exert anti-inflammatory effects by binding to the IL-6 receptor α (IL-6Rα), thus inhibiting inflammation [20]. This dual nature of IL-6 underscores the complexity of immune regulation and highlights the importance of contextual factors and receptor interactions in shaping its diverse roles within the immune response.

Interleukin-8 (IL-8): Interleukin-8 (IL-8), recognized primarily for its pro-inflammatory attributes, reveals a dual functionality, adding intricacy to its role in the immune response. While serving as a promoter of pro-inflammatory cytokines such as IL-1β and TNF-α in specific scenarios, IL-8 also demonstrates anti-inflammatory effects under different conditions [21]. For instance, IL-8 can inhibit the production of these cytokines in specific contexts, contributing to a nuanced regulatory role. The actions of dual-function cytokines, exemplified by IL-6 and IL-8, reflect the dynamic and context-dependent nature of immune responses and inflammation. The variability in their functions is intricately tied to the receptors they engage with and the specific circumstances surrounding their production [19]. Understanding these complexities adds depth to our comprehension of immune regulation and underscores the need for a nuanced approach to studying and manipulating cytokine functions within the immune system.

Regulation of cytokine expression

Cellular Sources of Cytokines

The origins of cytokines are diverse, and the oversight of cytokine expression encompasses various stages in gene expression. A broad spectrum of cells contributes to cytokine production, encompassing immune cells such as macrophages, T cells, B cells, endothelial cells, fibroblasts, and assorted cell types. The orchestration of cytokine expression transpires at multiple levels, including transcriptional, post-transcriptional, and translational phases. This intricate regulation involves a sophisticated interplay of transcription factors, RNA-binding proteins, and microRNAs. Notably, post-transcriptional regulation plays a pivotal role in governing cytokine expression, with the stability of cytokine mRNAs subject to meticulous control by RNA-binding proteins and microRNAs [22,23]. The induction, production, stimulation, and inhibition of diverse cytokines undergo stringent regulation to preserve immune homeostasis and mount appropriate responses to diverse stimuli. The intricate nature of cytokine redundancy and pleiotropy adds a layer of complexity to the precise characterization of cytokine signaling activities. Many cytokines exhibit cellular effects that may seem redundant within a specific cellular context and often manifest pleiotropic functions within an organism [22,23]. Consequently, the regulation of cytokine expression emerges as a complex and tightly controlled process, indispensable for the proper functioning of the immune system and the maintenance of immune homeostasis [22,23].

Transcriptional Regulation

Transcriptional regulation stands as a pivotal phase in controlling cytokine expression. The intricate orchestration of cytokine gene expression involves a cast of transcription factors, with notable contributors including nuclear factor kappa B (NF-κB), activator protein 1 (AP-1), and signal transducer and activator of transcription (STAT) proteins. These transcription factors specifically bind to defined DNA sequences within the promoter regions of cytokine genes, thereby dictating and overseeing their transcriptional processes [22,24,25]. This regulatory of cytokine gene transcription is multifaceted, entailing the intricate interplay of diverse signaling pathways and transcriptional regulators. Beyond the direct involvement of transcription factors, the expression of cytokines undergoes additional layers of control through epigenetic modifications. Processes like DNA methylation and histone modifications wield influence, impacting the accessibility of cytokine gene promoters to transcription factors [22,24,25]. The regulation of cytokine gene expression emerges as indispensable for properly functioning the immune system and maintaining immune homeostasis, underscoring its significance in orchestrating a balanced and effective immune response [22,24,25].

Post-transcriptional and Post-Translational Regulation

Post-transcriptional and post-translational regulation emerge as pivotal layers governing cytokine expression, involving intricate orchestration by RNA-binding proteins (RBPs) and microRNAs (miRNAs) to finely modulate mRNA stability, translation, and protein function [26]. Post-transcriptional regulation sets the stage in the 3' untranslated region (UTR) of mRNA molecules, acting as a hotspot for regulatory factors like microRNA targets [26]. This nuanced regulatory enables swift and transient responses to stimuli and external stress, allowing the immune system to adapt promptly to changing circumstances [26]. Moving into the domain of post-translational regulation, the influence of RNA-binding proteins (RBPs) takes center stage. These proteins wield their regulatory prowess by shaping cytokine production by modulating mRNA stability, translation initiation, and cytoplasmic localization [27]. Some RBPs act as navigators, directing associated mRNA molecules to specific cytoplasmic locales, thereby profoundly impacting cytokine production [27]. The intricacies of this regulation extend to the composition of the protein coat, which, in turn, influences cytoplasmic localization, stability, and translational competence. Sequence elements embedded in mRNA molecules play a crucial role in recruiting proteins that, in turn, influence mRNA processing and stability [27]. A deficiency in the delicate control mechanisms of post-transcriptional and post-translational regulation can have profound consequences. It may result in heightened and sustained production of pro-inflammatory mediators, thereby contributing to the pathogenesis of various diseases [27]. Thus, unraveling the intricacies of these regulatory mechanisms becomes paramount for comprehending the nuanced nature of cytokine action in diverse physiological and pathological processes.

Immunomodulatory therapies in sepsis

Overview of Current Treatments

The comprehensive care of patients with sepsis involves a multifaceted approach, as highlighted by the integration of supportive care strategies. As outlined in the literature [28], this approach encompasses essential elements such as timely administration of antibiotics, source control, resuscitation measures, and organ support. By addressing septic individuals' immediate and critical needs, this holistic strategy forms a supportive framework crucial for facilitating recovery. Exploring immunomodulatory agents, particularly the investigation of promising candidates like IL-7, adds a layer of sophistication to sepsis treatment [29]. However, the outcomes of clinical trials focused on immunosuppressive strategies have been less than optimal, emphasizing the need for a more personalized and nuanced treatment paradigm [28]. This recognition underscores the evolving landscape of sepsis management, prompting a shift toward precision immunotherapy.

Innovative approaches, such as precision immunotherapy, signify a paradigm shift in sepsis treatment. Tailoring interventions based on the unique immune status of each patient have shown promise, exemplified by the successful restoration of HLA-DR expression and monocyte function through interferon-gamma application [30]. This precision-focused strategy marks a significant advancement, aligning with the broader trend in healthcare toward personalized and targeted treatments. Strategic monitoring of the immune status of septic patients emerges as a key component in pursuing enhanced therapeutic outcomes. By systematically evaluating immune responses, opportunities for targeted interventions arise, potentially improving the efficacy of immunomodulation in sepsis [30]. Despite the ongoing quest for a singular adjunctive treatment for sepsis, the absence of a universally established therapy underscores the complexity of immune responses in this condition [28]. This emphasizes the critical need for an enriched understanding of sepsis immunology, coupled with the application of precision medicine approaches that tailor interventions to the unique characteristics of individual patients [28,30].

Challenges in Targeting Cytokine Storm

The immune response in sepsis is characterized by its inherent complexity, involving many cells and molecules. This intricate network of immune processes presents a formidable challenge in developing targeted therapeutic interventions, as highlighted in the literature [13]. The multifaceted nature of the immune response complicates the task of identifying precise targets for intervention, making it imperative to unravel the intricacies of this complex system. A critical limitation in current sepsis research is the lack of precise immunological monitoring throughout therapeutic development. Clinical trials targeting cytokine production or effects have encountered setbacks due to the absence of accurate monitoring tools [30]. This deficiency hampers the ability to thoroughly assess the efficacy and safety of potential immunotherapies, hindering progress in the field.

The delicate balance between pro-inflammatory and anti-inflammatory responses further adds to the challenges in sepsis treatment. Sepsis patients often exhibit an intricate imbalance in these responses, complicating the development of therapies capable of effectively modulating both types. This duality poses a considerable hurdle in devising treatment strategies that address the diverse aspects of the immune response in sepsis [30]. Notably, a historical focus on pro-inflammatory pathways in sepsis has led to limited success with anti-inflammatory therapies. Therapeutic strategies have often neglected the immune suppression induced by sepsis, resulting in constrained success in achieving desired therapeutic outcomes [29]. This underscores the necessity for a more comprehensive understanding of the immune response spectrum in sepsis to inform targeted interventions.

Identifying the optimal timing for intervention is paramount in sepsis treatment, requiring a delicate balance to avoid exacerbating inflammation or compromising immune function. The challenge lies in determining the opportune moment for intervention, emphasizing the importance of temporal precision in therapeutic strategies [30]. Despite these formidable challenges, an ongoing exploration of novel therapeutic approaches is underway, focusing on immunomodulatory agents and innovative strategies to enhance immune cell function. These endeavors hold promise in navigating the intricate landscape of immune responses in sepsis and potentially improving patient outcomes [29].

Emerging Therapeutic Approaches

The realm of sepsis treatment is expanding to embrace diverse and innovative therapeutic approaches, including herbal medicine, immunonutrition, targeted supportive therapies, immunostimulatory therapy, personalized immunotherapy, and immunomodulatory agents. Herbal medicines have shown promise in modulating immune responses and mitigating inflammation in sepsis patients, representing a diverse avenue for potential therapeutic interventions [31]. Immunonutrition, involving the administration of specific nutrients, emerges as a targeted approach to augmenting the immune response and contributing to improved outcomes for sepsis patients. Targeted supportive therapies focus on supplying essential nutrients, oxygen, and other resources to empower the body's natural ability to combat infection, thereby enhancing the overall resilience of the immune system during sepsis [31]. Immunostimulatory therapy strategically employs cytokines, growth factors, and immune checkpoint inhibitors to amplify the immune response in individuals grappling with sepsis, aiming to invigorate the immune system's effectiveness against infection [32].

Personalized immunotherapy, tailoring treatments to individual patients based on their unique immune status and response nuances, stands out as a targeted and individualized strategy for combating sepsis [28]. Meanwhile, ongoing exploration involves investigating immunomodulatory agents and innovative therapeutics designed to enhance the functionality of immune cells, recalibrating and optimizing the immune response in septic individuals [29]. A proactive approach involves continuously monitoring the immune status in septic patients, providing valuable insights for targeted interventions in immunomodulation [30]. This strategy enables a more nuanced understanding of dynamic immune responses, potentially leading to more successful outcomes in treating sepsis. While these emerging therapeutic approaches hold substantial promise for advancing sepsis treatment, their efficacy and safety necessitate further research and rigorous clinical validation. Determining the optimal strategies for individual patients requires a deeper understanding of the nuanced interactions within the immune system and the specific contexts of septic conditions [31]. As research progresses, these diverse approaches offer hope for more effective and tailored interventions in the complex landscape of sepsis treatment.

Biomarkers for cytokine storm in sepsis

Identification of Biomarkers

Pro-inflammatory cytokines, including interleukin-1β (IL-1β), CCL4, CCL5, and CXCL10, have emerged as potential biomarkers with significant implications for diagnosing sepsis and assessing the severity of cytokine storms [33]. These molecular indicators are crucial in elucidating the inflammatory milieu associated with septic conditions, offering valuable insights into the dynamic immune responses during such critical states. In the context of sepsis and COVID-19, research has revealed distinct alterations in biomarkers of inflammation and cytokine storms. This emphasizes the necessity for specific and tailored biomarkers finely attuned to the nuanced characteristics of different inflammatory conditions [34]. The unique inflammatory signatures observed in each condition underscore the need for precise discrimination in biomarker selection.

Despite the promise exhibited by various pro-inflammatory cytokines as potential biomarkers for cytokine storms, their translation into effective clinical treatments has encountered substantial challenges. Clinical trials targeting cytokine production or effects have yet to yield successful treatments, underscoring the imperative for more comprehensive and precise biomarker identification and validation [13]. Bridging the gap between promising biomarkers and clinical efficacy remains a formidable task, necessitating continued efforts in refining biomarker selection and validation processes. Cytokines such as MIP-1α and TNF-α have been proposed as potential biomarkers for cytokine storms induced by infectious diseases, including sepsis. However, their utility in clinical settings requires thorough validation through further research [35]. Identifying reliable biomarkers for cytokine storms in sepsis is an intricate and evolving process. While specific pro-inflammatory cytokines exhibit promise as potential biomarkers, their effective translation into clinical treatments remains a formidable challenge. Rigorous research is indispensable for confirming their utility in diagnosing and managing cytokine storms in sepsis patients, paving the way for more targeted and effective therapeutic strategies.

Diagnostic and Prognostic Significance

Exploring the diagnostic and prognostic implications of cytokine storms in sepsis is a rapidly evolving area of research. Cytokine storm, a crucial element in the pathogenesis of sepsis, precipitates an uncontrolled inflammatory response, contributing to multiple organ dysfunctions and, ultimately, mortality [13]. Specific pro-inflammatory cytokines, including IL-1β, CCL4, CCL5, and CXCL10, have emerged as potential diagnostic markers for sepsis and critical indicators of cytokine storm severity [33]. Regarding diagnostic significance, pro-inflammatory cytokines provide a distinct marker for discerning sepsis from other non-septic conditions, given their specific elevation in sepsis patients [33]. Notably, the presence of a cytokine storm can serve as an early indicator of sepsis severity, offering valuable insights for timely diagnosis and the initiation of treatment [13]. Moving on to prognostic significance, the level of cytokine storm becomes a predictive factor for both the severity of sepsis and the clinical outcomes of affected individuals [13]. Early identification of cytokine storms is crucial in risk stratification, providing a foundation for tailoring therapeutic interventions based on the observed severity [12]. Despite the promising potential of these cytokines as biomarkers, translating these findings into effective clinical treatments has encountered significant challenges. Rigorous research efforts are indispensable to validate the utility of these biomarkers in diagnosing and managing cytokine storms in sepsis patients [13]. The journey toward clinical applicability requires continued exploration to bridge the gap between promising biomarkers and tangible advancements in the management of sepsis.

Limitations and Future Directions

Several challenges and limitations surround the diagnostic and prognostic use of pro-inflammatory cytokines in sepsis, raising important considerations for future research and clinical applications. Firstly, the lack of specificity poses a significant hurdle, as specific pro-inflammatory cytokines may not be exclusive to sepsis and can also be elevated in other inflammatory conditions or infections [33]. Moreover, the variability in cytokine levels among patients adds another layer of complexity. The significant variations make it challenging to establish a universal cutoff value for diagnosing sepsis or assessing the severity of cytokine storms [36]. Additionally, the intricate regulatory mechanisms governing cytokine production, involving transcription factors, RNA-binding proteins, and microRNAs, contribute to the challenge of identifying a straightforward relationship between cytokine levels and disease severity [13]. The difficulty in translating promising findings into clinical treatments is underscored by the fact that despite identifying potential cytokine biomarkers, clinical trials targeting cytokine production or effects have yet to yield successful treatments [36]. This emphasizes the need for more comprehensive and precise biomarker identification and validation. To address these limitations, future research directions could explore innovative approaches. One avenue involves investigating cytokine signatures or gene expression profiling to enhance the diagnostic accuracy and prognostic significance of cytokine storms in sepsis [33]. Another promising direction is the exploration of personalized medicine approaches, wherein individual patient data and immune status are considered to tailor treatments and monitor cytokine storms more effectively [36]. Developing new therapeutic strategies focusing on the endothelium as a source and target of cytokines holds promise for more effectively managing cytokine storms in sepsis [13]. These avenues of exploration signify potential breakthroughs in overcoming the existing challenges and advancing the clinical utility of cytokines in sepsis diagnosis and treatment.

Experimental models and clinical studies

Animal Models for Studying Cytokine Storm

Animal models have played a pivotal role in advancing our understanding of cytokine storms, particularly in sepsis. These models have provided valuable insights into the pathophysiological mechanisms and potential therapeutic strategies. Several noteworthy findings and approaches from the literature underscore the significance of animal models in cytokine storm research. One notable model is the Experimental Macrophage Activation Syndrome Model, which has yielded evidence supporting the role of Th17 cells in driving a cytokine storm, particularly in the context of secondary hemophagocytic lymphohistiocytosis (HLH) [15]. This model has contributed significantly to unraveling the intricate cellular interactions underlying the development of cytokine storms. The study of infectious diseases and sepsis has been a focal point, where animal models have been instrumental in mimicking conditions seen in severe infections. The severity of infections can trigger an activation cascade, resulting in an autoamplifying cytokine storm. These models have provided a controlled environment to study the complex immune responses associated with severe infections [13]. In therapeutic approaches, animal models have been employed to evaluate potential strategies, such as apoptotic cell therapy targeted at cytokine storms linked to acute severe sepsis. Insights gained from these studies have demonstrated the rebalancing effects of specific therapies on cytokine storms, offering valuable information for potential clinical applications [12]. Additionally, animal models have been crucial in biomarker research related to cytokine storms and inflammation in sepsis. These investigations have led to the identification of potential biomarkers and an enhanced understanding of their diagnostic and prognostic significance [34]. By mimicking sepsis conditions, these models have provided a controlled environment for biomarker research, aiding in translating findings to clinical settings. Animal models have proven to be invaluable tools in the study of cytokine storms in sepsis. They have enhanced our understanding of the pathophysiological mechanisms and offered insights into potential therapeutic strategies and the identification of biomarkers. These models continue to be instrumental in developing novel treatment approaches for cytokine storms associated with sepsis.

Clinical Studies on Cytokine Storm in Sepsis

Clinical studies on cytokine storm in sepsis encounter notable challenges, including the lack of specificity in cytokine markers, variations in cytokine levels among patients, and the intricate translation of findings into clinically effective treatments. One focus of clinical investigations involves infections as triggers for cytokine storms. Cytokine storms can arise from naturally occurring microbial infections, with the severity leading to an autoamplifying cytokine storm. Clinical studies have concentrated on unraveling the immune responses to various infections and identifying potential triggers for cytokine storm development [13]. Clinical trials have been undertaken in therapeutic approaches to assess potential strategies for addressing cytokine storms in sepsis, such as apoptotic cell therapy. These trials have provided valuable insights into the rebalancing effects of specific therapies on the cytokine storm, shedding light on their potential clinical applications [15]. Another avenue of clinical research involves biomarker exploration. Studies have been conducted to investigate biomarkers associated with cytokine storm and inflammation in sepsis, identifying potential biomarkers and enhancing our understanding of their diagnostic and prognostic significance [34]. Clinical studies have delved into the role of Th17 cells in cytokine storm, particularly in the context of secondary Hemophagocytic Lymphohistiocytosis (HLH). Evidence from these studies suggests that Th17 cells can drive a cytokine storm in this specific context [15]. While clinical studies on cytokine storms in sepsis face challenges, they have yielded valuable insights into the pathophysiology of cytokine storms, potential therapeutic strategies, and the identification of biomarkers. Overcoming these challenges will require further research to develop effective treatments for cytokine storm in sepsis, ultimately advancing our ability to manage this critical condition.

Gaps in Current Research

The current research on sepsis immunology and immunomodulatory therapies has identified several gaps that must be addressed with high priority. Understanding the immunological mechanisms underlying sepsis, including the complex interplay of pro- and anti-inflammatory responses and the factors contributing to immunoparalysis [30,37]. Translating emerging non-conventional immunomodulatory approaches, such as herbal medicine, immunonutrition, and targeted supportive therapies, into everyday clinical practice through further research and stringent clinical validation [31]. Developing precise immunological monitoring methods to guide immunomodulatory therapies in sepsis may lead to more successful outcomes [30]. Identifying specific biomarkers that can accurately diagnose and assess the severity of cytokine storm in sepsis and predict patient outcomes [38]. Addressing these gaps in current research will be essential for advancing the understanding of sepsis immunology and developing more effective immunomodulatory therapies for managing sepsis.

## Conclusions

In conclusion, this comprehensive review has delved into the intricate dynamics of sepsis, shedding light on the pivotal roles of chemokines and cytokines in orchestrating the cytokine storm. The journey began with exploring sepsis stages, elucidating the progression from infection to severe sepsis and septic shock. Our examination of the immune response in sepsis highlighted the delicate balance disrupted during this dysregulated cascade, with immune cells contributing to tissue damage rather than protection. Focusing on chemokines, we elucidated their diverse subfamilies, receptors, and functions in the context of sepsis, revealing their profound impact on immune cell migration and activation. The culmination of these insights underscores the significance of the cytokine storm in sepsis pathology. The implications for clinical practice are substantial, suggesting avenues for targeted diagnostics and therapeutic interventions. The need for clinicians to stay abreast of emerging research is evident as the field evolves. Furthermore, this review has identified areas for future investigation, including searching for novel biomarkers, a deeper understanding of cytokine regulation, and pursuing personalized medicine approaches. As we navigate the complex landscape of sepsis, armed with a nuanced understanding of chemokines and cytokines, the potential for transformative advancements in research and clinical care emerges, holding promise for improved outcomes in the challenging realm of sepsis management.

## References

[1] (2023). Sepsis. https://www.who.int/news-room/fact-sheets/detail/sepsis.

[2] Stearns-Kurosawa DJ, Osuchowski MF, Valentine C, Kurosawa S, Remick DG (2011). The pathogenesis of sepsis. Annu Rev Pathol.

[3] Jarczak D, Kluge S, Nierhaus A (2021). Sepsis—pathophysiology and therapeutic concepts. Front Med (Lausanne).

[4] Dunkin MA, Garceau A (2023). What Is Sepsis or Septicemia (Blood Infection)?. http://webmd.com/a-to-z-guides/sepsis-septicemia-blood-infection.

[5] Wiersinga WJ, Leopold SJ, Cranendonk DR, van der Poll T (2014). Host innate immune responses to sepsis. Virulence.

[6] Schulte W, Bernhagen J, Bucala R (2013). Cytokines in sepsis: potent immunoregulators and potential therapeutic targets--an updated view. Mediators Inflamm.

[7] Hughes CE, Nibbs RJ (2018). A guide to chemokines and their receptors. FEBS J.

[8] Li H, Wu M, Zhao X (2022). Role of chemokine systems in cancer and inflammatory diseases. MedComm (2020).

[9] (2023). Chemokine types. https://www.news-medical.net/health/Chemokine-Types.aspx.

[10] Bennett LD, Fox JM, Signoret N (2011). Mechanisms regulating chemokine receptor activity. Immunology.

[11] de Pablo R, Monserrat J, Prieto A, Álvarez-Mon M (2013). Role of circulating soluble chemokines in septic shock. Med Intensiva.

[12] Karbian N, Abutbul A, El-Amore R, Eliaz R, Beeri R, Reicher B, Mevorach D (2020). Apoptotic cell therapy for cytokine storm associated with acute severe sepsis. Cell Death Dis.

[13] Chousterman BG, Swirski FK, Weber GF (2017). Cytokine storm and sepsis disease pathogenesis. Semin Immunopathol.

[14] Jarczak D, Nierhaus A (2022). Cytokine storm—definition, causes, and implications. Int J Mol Sci.

[15] Fajgenbaum DC, June CH (2020). Cytokine storm. N Engl J Med.

[16] (2023). Pro-inflammatory cytokines overview. https://www.thermofisher.com/in/en/home/life-science/cell-analysis/cell-analysis-learning-center/immunology-at-work/proinflammatory-cytokines-overview.html.

[17] Zhang JM, An J (2007). Cytokines, inflammation, and pain. Int Anesthesiol Clin.

[18] De Caterina R, Libby P, Peng HB (1995). Nitric oxide decreases cytokine-induced endothelial activation. Nitric oxide selectively reduces endothelial expression of adhesion molecules and proinflammatory cytokines. J Clin Invest.

[19] Luheshi NM, Rothwell NJ, Brough D (2009). Dual functionality of interleukin-1 family cytokines: implications for anti-interleukin-1 therapy. Br J Pharmacol.

[20] (2023). Anti-inflammatory cytokines list. https://www.sinobiological.com/resource/cytokines/all-anti-inflammatory-cytokines.

[21] Brat DJ, Bellail AC, Van Meir EG (2005). The role of interleukin-8 and its receptors in gliomagenesis and tumoral angiogenesis. Neuro Oncol.

[22] Palanisamy V, Jakymiw A, Van Tubergen EA, D'Silva NJ, Kirkwood KL (2012). Control of cytokine mRNA expression by RNA-binding proteins and microRNAs. J Dent Res.

[23] Jiang P, Zhang Y, Ru B (2021). Systematic investigation of cytokine signaling activity at the tissue and single-cell levels. Nat Methods.

[24] Taniguchi T (1988). Regulation of cytokine gene expression. Annu Rev Immunol.

[25] Holloway AF, Rao S, Shannon MF (2002). Regulation of cytokine gene transcription in the immune system. Mol Immunol.

[26] Khabar KS (2014). Post-transcriptional control of cytokine gene expression in health and disease. J Interferon Cytokine Res.

[27] Anderson P (2008). Post-transcriptional control of cytokine production. Nat Immunol.

[28] Peters van Ton AM, Kox M, Abdo WF, Pickkers P (2018). Precision immunotherapy for sepsis. Front Immunol.

[29] Hutchins NA, Unsinger J, Hotchkiss RS, Ayala A (2014). The new normal: immunomodulatory agents against sepsis immune suppression. Trends Mol Med.

[30] Kox WJ, Volk T, Kox SN, Volk HD (2000). Immunomodulatory therapies in sepsis. Intensive Care Med.

[31] Slim MA, Turgman O, van Vught LA, van der Poll T, Wiersinga WJ (2023). Non-conventional immunomodulation in the management of sepsis. Eur J Intern Med.

[32] Marques A, Torre C, Pinto R, Sepodes B, Rocha J (2023). Treatment advances in sepsis and septic shock: modulating pro- and anti-inflammatory mechanisms. J Clin Med.

[33] Frimpong A, Owusu ED, Amponsah JA (2022). Cytokines as potential biomarkers for differential diagnosis of sepsis and other non-septic disease conditions. Front Cell Infect Microbiol.

[34] Titov L, Trusevich M, Amvrosieva T, Belskaya I, Gorbich Y, DuBuske L (2022). Biomarkers of cytokine storm and inflammation in sepsis, post-covid-19 patients and covid-19 vaccinated individuals. Ann Allergy Asthma Immunol.

[35] Tang XD, Ji TT, Dong JR (2021). Pathogenesis and treatment of cytokine storm induced by infectious diseases. Int J Mol Sci.

[36] Cho SY, Choi JH (2014). Biomarkers of sepsis. Infect Chemother.

[37] Rubio I, Osuchowski MF, Shankar-Hari M (2019). Current gaps in sepsis immunology: new opportunities for translational research. Lancet Infect Dis.

[38] Bermejo-Martin JF, García-Mateo N, Motos A (2023). Effect of viral storm in patients admitted to intensive care units with severe COVID-19 in Spain: a multicentre, prospective, cohort study. Lancet Microbe.

